# Progressive encephalomyelitis with rigidity and myoclonus after thymectomy in a woman with myasthenia gravis

**DOI:** 10.3389/fimmu.2025.1563700

**Published:** 2025-04-16

**Authors:** Lixia Qin, Weiqian Yan

**Affiliations:** ^1^ Department of Neurology, The Second Xiangya Hospital, Central South University, Changsha, Hunan, China; ^2^ Clinical Medical Research Center for Stroke Prevention and Treatment of Hunan Province, Department of Neurology, the Second Xiangya Hospital, Central South University, Changsha, China

**Keywords:** PERM, stiff-person syndrome, GAD65, GlyR, myasthenia gravis

## Abstract

Progressive encephalomyelitis with rigidity and myoclonus (PERM), part of the spectrum of stiff-person syndrome (SPS), is a rare neurological disorder characterized by axial and limb rigidity, painful muscle spasms, hyperekplexia, brainstem signs, and autonomic dysfunction. Here, we present the case of a 44-year-old woman with a history of myasthenia gravis (MG) who had previously undergone a thymectomy. She presented with a 20-day history of startle-induced episodes of generalized rigidity and painful spasms affecting her face, trunk, and limbs. Her symptoms began gradually, initially with numbness and pain on the right side of her face, followed by sudden episodes of myoclonus and jerking, predominantly in axial muscles, triggered by auditory stimuli and light touch. Laboratory tests revealed positive serum and cerebrospinal fluid (CSF) antibodies, including glutamic acid decarboxylase (GAD65) antibodies (titer of 1:30), α1-subunit of the glycine receptor (GlyR) antibodies (titer of 1:10), acetylcholine receptor (AChR) antibodies (>20 nmol/L), and titin antibodies (18.6 U/mL). Extensive testing ruled out other autoantibodies and tumors, leading to a diagnosis of PERM. The patient was treated with intravenous methylprednisolone, oral clonazepam, and tacrolimus, which resulted in significant clinical improvement. A 2-year follow-up demonstrated sustained recovery, accompanied by a decrease in GAD65 antibody titers. In conclusion, PERM can occur in patients with MG, even after thymectomy. Given that most patients respond well to immunosuppressive therapies, timely diagnosis and intervention are crucial.

## Introduction

Stiff-person syndrome (SPS) is a neurological disorder characterized by fluctuating muscle rigidity and painful spasms, which can occur spontaneously or be triggered by various stimuli (1–3). The underlying immune mechanisms involve target proteins primarily expressed at inhibitory synapses. Six autoantigens have been identified, namely, glutamic acid decarboxylase (GAD65), the α1-subunit of the glycine receptor (GlyR), amphiphysin, gephyrin, dipeptidyl peptidase-like protein 6 (DPPX), and the γ-aminobutyric acid-A (GABA-A) receptor (GABAaR). In 1976, Whiteley et al. first reported two cases of encephalomyelitis characterized by muscular rigidity and stimulus-sensitive muscular spasms, distinguishing them from classic SPS (4). Progressive encephalomyelitis with rigidity and myoclonus (PERM) represents a more severe clinical spectrum of SPS, featuring hyperekplexia, myoclonus, and dysfunctions of the brainstem, pyramidal tracts, sensory system, and autonomic nervous system (5). Although PERM is primarily associated with GlyR antibodies, some patients also harbor anti-GAD antibodies or have a coexistence of GlyR and GAD antibodies. Herein, we present a case of PERM positive for both GlyR and GAD antibodies, occurring post-thymectomy in a patient with myasthenia gravis (MG).

## Case description

A 44-year-old woman presented in December 2020 with a 20-day history of startle-induced episodes of generalized rigidity and painful spasms affecting her face, trunk, and limbs. Her symptoms began insidiously and progressed gradually. Initially, she experienced numbness and pain in the right side of her face, followed by sudden episodes of myoclonus and jerks, predominantly in axial muscles, triggered by auditory stimuli and light touch. Each paroxysmal episode lasted a few seconds, resolved spontaneously, and recurred dozens of times per day. Due to the frequency of these attacks, she was unable to walk independently. Her sleep was restless, and she preferred to keep her eyes closed due to diplopia. Additional symptoms included a weak voice, painful shoulder joints, and difficulty rolling over or combing her hair. Moreover, the patient experienced pronounced dry mouth and excessive sweating. No significant symptoms of urinary retention or constipation were reported. She had been hospitalized 8 months prior due to similar episodes, including numbness in the right side of her face, myoclonic jerks in her right face and limbs triggered by light touch, and leftward gaze deviation. At that time, her screening workup for infections [e.g., peripheral blood cell count, C-reactive protein, procalcitonin, erythrocyte sedimentation rate, cerebrospinal fluid (CSF) analysis, HIV, and venereal disease research laboratory testing), endocrine function (e.g., thyroid function tests), and metabolic parameters (e.g., blood glucose, lactic acid, uric acid) were all normal or negative. Autoantibodies associated with central nervous system demyelination [aquaporin-4 (AQP4), myelin oligodendrocyte glycoprotein (MOG), myelin basic protein (MBP)], encephalomyelitis/encephalitis (IgLON5, DPPX, DRD2, mGluR5, mGluR1, neurexin-3α, NMDA, AMPA1, AMPA2, LGI1, GABAb, CASPR2), and gangliosides (GM1, GM2, GM3, GM4, GD1a, GD1b, GD2, GD3, GQ1b, GT1a, GT1b, sulfatide) were not detected in either serum or CSF. Brain magnetic resonance imaging (MRI) performed in March 2020, including T1-weighted imaging (T1-WI), T2-weighted imaging (T2-WI), fluid-attenuated inversion recovery (FLAIR), contrast enhancement, and diffusion-weighted imaging (DWI), was normal. Symptoms had almost completely resolved with temporary corticosteroid treatment. The patient had a history of MG associated with thymoma, diagnosed 8 years ago. At the time of diagnosis, she presented with bilateral ptosis, diplopia, and generalized limb weakness, without evidence of bulbar palsy or respiratory distress. She underwent a thymectomy followed by chemotherapy and symptomatic treatment, leading to a gradual resolution of symptoms and eventual full recovery. Since then, she has been maintained on pyridostigmine bromide (60 mg/day). No family history of neurological diseases was reported.

On examination, she exhibited a painful facial expression and abnormal posture, with a crooked back due to back pain. She had a weak voice but no dysarthria. Gaze-evoked nystagmus was noted on lateral gaze, particularly rightward gaze. Paroxysmal limb rigidity was prominent, accompanied by a positive Babinski response. The patient exhibits significantly increased muscle tone in all four limbs and the trunk, accompanied by prominent pain. During episodes of myoclonus triggered by auditory or visual stimuli, the pain intensifies markedly, causing severe distress and a sense of impending doom. Tendon reflexes were normal, and finger-to-nose and heel-to-shin testing were unremarkable. The remainder of the neurological examination was unremarkable (**Videos 1**, **2**).

**Figure 1:**
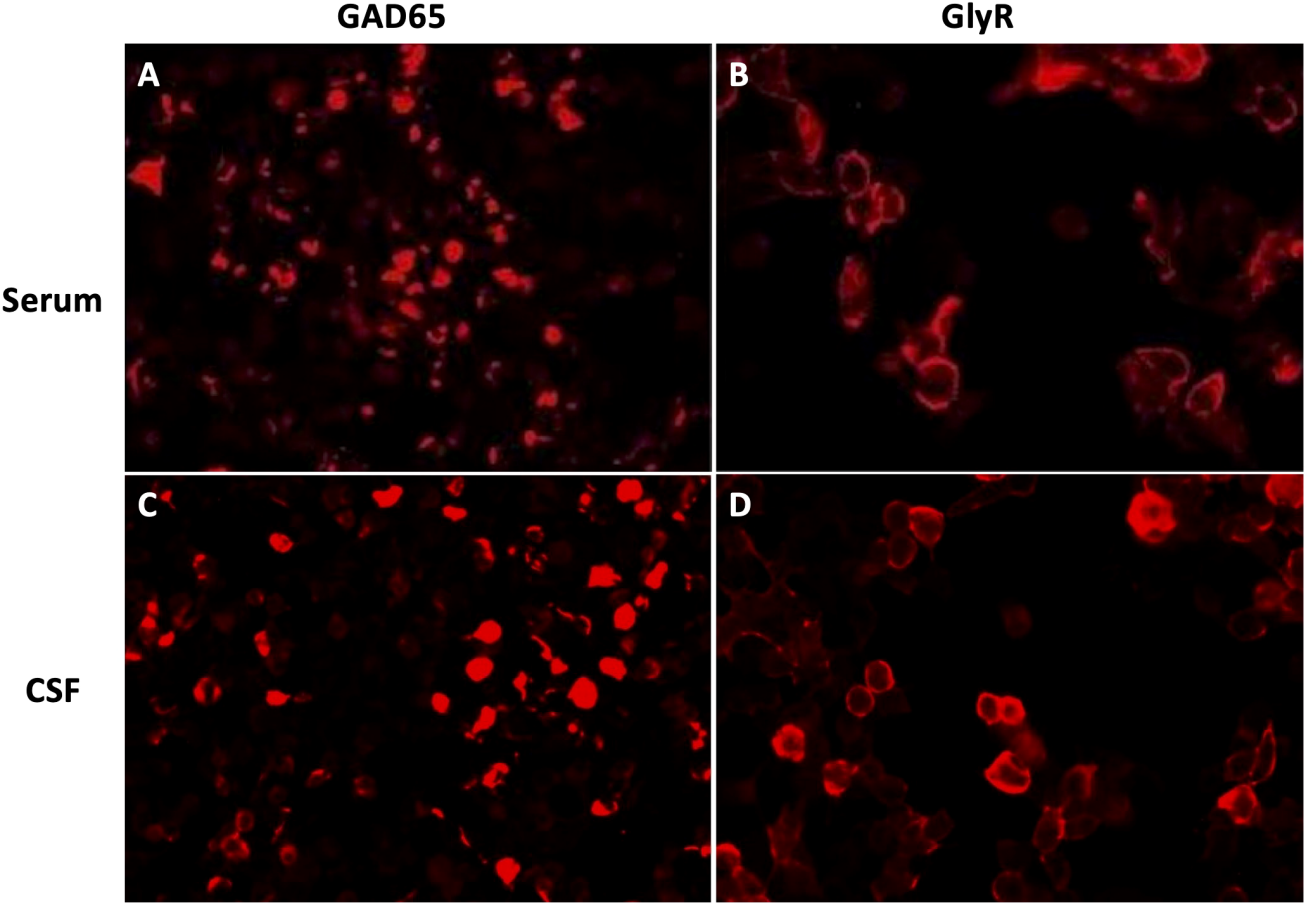
GAD65 **(A, C)** and GlyR **(B, D)** antibodies were assessed by immunofluorescence in both serum **(A, B)** and CSF **(C, D)** on admission (January 2021). CSF, cerebrospinal fluid.

**Figure 2:**
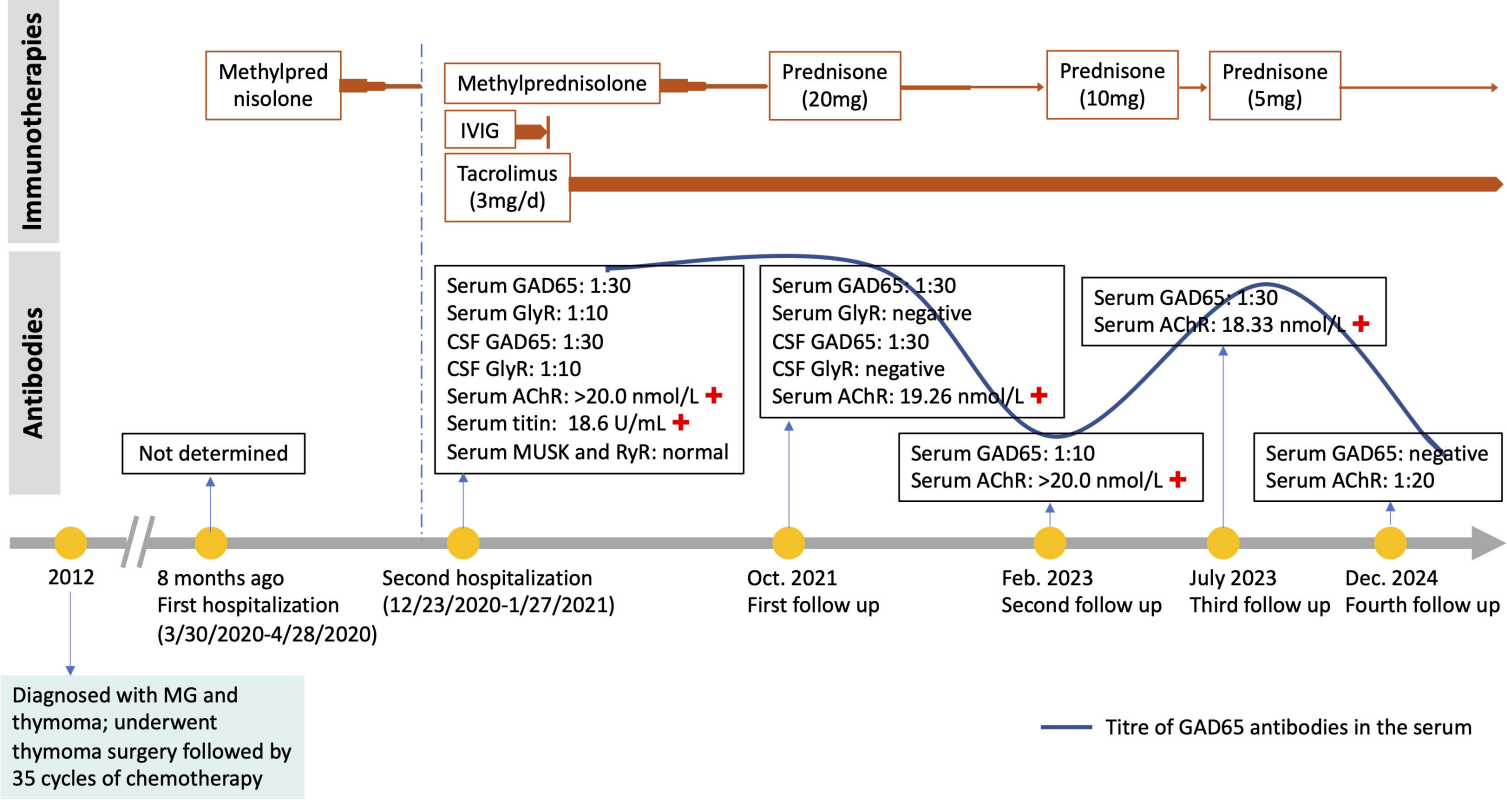
A timeline of events. +, positive; IVIG, intravenous immunoglobulin; CSF, cerebrospinal fluid.

Routine laboratory tests, including thyroid function, serum ammonia, lactic acid, creatine kinase, ceruloplasmin, and urine copper levels, were unremarkable. CD8-positive T-lymphocyte levels were elevated, while CD4-positive T lymphocytes were decreased. Repeated CSF white blood cell count, protein, glucose, and chloride levels were all within normal limits, with no evidence of infection. However, intrathecal synthesis of IgG was detected. Testing for acetylcholine receptor (AChR) antibodies and titin antibodies was strongly positive (>20.0 nmol/L and 18.6 U/mL, respectively). Tests for antithyroid peroxidase antibodies (32.9 IU/mL; normal: <60 IU/mL) and antithyroglobulin antibodies (<15 IU/mL; normal: <60 IU/mL) yielded negative results. Antineuronal antibodies (Hu, Yo, Ri, CV2, amphiphysin, Ma2/Ta) were negative. Repeated autoantibodies associated with central nervous system demyelination, encephalomyelitis/encephalitis, and gangliosides were not detected in either serum or CSF. Extensive tumor screening was performed, but no malignancies were found. Brain and spinal MRI (T1-WI, T2-WI, FLAIR, contrast enhancement, and DWI) showed no abnormalities. Moreover, imaging studies of the shoulder, thymus, chest, and abdomen, as well as electromyography (EMG), were all normal. Both anti-GAD65 (titer of 1:30) and anti-GlyR (titer of 1:10) antibodies were detected in both serum and CSF (**Figure 1**). No other autoantibodies associated with the SPS spectrum were identified. The patient was treated with intravenous immunoglobulin for 5 days (400 mg/kg/day) and intravenous methylprednisolone (1,000 mg/day) for 5 days followed by an oral prednisone taper, tacrolimus (3 mg/day), and clonazepam (7.5 mg/day). In the early post-discharge period, the patient’s increased muscle tone had not completely resolved. However, significant improvement was noted in diplopia and dizziness, and the patient’s mental state was positive, with a willingness to attempt walking. With the assistance of mobility aids and family support, the patient was able to walk slowly. As symptoms gradually improved, the patient’s gait became notably more stable. At the 9-month follow-up, the patient was observed to walk independently on flat ground with only the aid of a cane. At that time, serum GlyR antibodies were undetectable. However, serum and CSF anti-GAD65 antibodies (titer of 1:30) remained detectable. Over a 2-year follow-up period, she experienced no relapses and maintained a good recovery. Her gait had returned to near-normal. A repeat serum anti-GAD65 antibodies test showed a titer of 1:10. At the 4-year follow-up, serum anti-GAD65 antibodies had converted to negative. A timeline displaying relevant data on antibody titers and immunotherapy is shown in **Figure 2**. More information about antibody titers is displayed in **Table 1**.

**Table 1:** Antibody titers at different time points.

Time	Serum GAD65	Serum GlyR	CSF GAD65	CSF GlyR	Serum AChR	Serum titin	Serum MUSK	Serum RyR
1/12/2021	1:30^a^	1:10^a^	1:30^a^	1:10^a^	>20^b^ **↑**	18.6^b^ **↑**	–	–
3/11/2021	1:30^a^	–	ND	ND	19.26^b^ **↑**	ND	ND	ND
10/22/2021	1:30^a^	–	1:30^a^	–	10.17^b^ **↑**	–	–	–
2/28/2023	1:10^a^	ND	ND	ND	>20^b^ **↑**	ND	ND	ND
7/29/2023	1:30^a^	ND	ND	ND	18.33^b^ **↑**	–	–	–
12/10/2024	–	ND	ND	ND	1:10^a^	ND	ND	ND

ND, not determined; −, negative or normal; ↑, increased.

a Cell-based assay (CBA).

b Enzyme-linked immunosorbent assay (ELISA).

## Discussion

PERM shares similarities with SPS, including rigidity, stimulus-sensitive spasms, myoclonus, hyperekplexia, and autonomic dysfunction. However, PERM is distinguished by additional brainstem or other neurological deficits (4, 5). Generally, PERM is more severe and progressive than SPS. Most patients present in their fifth or sixth decade of life with a gradual onset and a relapsing-remitting course, exhibiting prominent brainstem dysfunction and dysautonomia alongside SPS symptoms. While most PERM patients have autoantibodies targeting GlyR, some may also exhibit coexisting GlyR and GAD antibodies (5–7). In our case, blood and CSF tests revealed the presence of anti-GAD65 antibodies (titer of 1:30) and anti-GlyR antibodies (titer of 1:10) with no other autoantibodies associated with the SPS spectrum detected. The final clinical classification was primarily based on the criteria proposed by Meinck and Thompson (2002) and Espay and Chen (2006), which define PERM as involving brainstem dysfunction in addition to the typical axial or limb rigidity seen in various forms of SPS (1, 8).

GlyRs belong to the superfamily of ligand-gated ion channels and are pentameric proteins consisting of two α and three β subunits (9). Activation of GlyRs by glycine leads to chloride ion influx into neurons, resulting in membrane hyperpolarization and reduced excitation. The detection of GlyR antibodies aids in diagnosing patients presenting symptoms such as ocular motor and other brainstem dysfunctions, hyperekplexia, stiffness, rigidity, myoclonus, and spasms. In this case, anti-GlyR antibodies were detected in both serum and CSF. During follow-up, we observed that with the recovery of symptoms, the titer of anti-GlyR antibodies in blood decreased until it was undetectable, while the CSF GlyR antibody detection was not performed as the patient declined to undergo a lumbar puncture. Anti-GlyR antibodies are commonly associated with SPS, and their detection in serum or CSF provides further immunological evidence supporting an SPS diagnosis.

GlyR antibodies target cell surface antigens, whereas GAD65 antibodies target intracellular antigens. The coexistence of both GlyR and GAD antibodies suggests that neuronal surface antibodies, potentially targeting different sites, may influence the clinical phenotype of GAD-associated diseases. Compared to patients with GAD65 antibodies alone, those with GlyR antibodies tended to have better outcomes (10). In general, patients with both GAD65 and GlyR antibodies demonstrate favorable prognoses. In our case, the patient remained relapse-free following successful treatment.

Interestingly, PERM developed in this patient following thymectomy for MG. MG is frequently associated with other autoimmune conditions, including autoimmune thyroid disease, autoimmune encephalitis, inflammatory myopathies, Sjögren’s syndrome, and systemic lupus erythematosus (11, 12). Recent reports have documented cases of MG coexisting with SPS or PERM (13–17). To date, two cases of PERM and MG with anti-GlyR/GAD and anti-AChR antibodies associated with thymoma have been reported (15, 18), whose clinical characteristics and outcomes are summarized in **Table 2**. Given the strong association between MG, PERM, and thymomas, this finding is not unexpected. In thymoma patients, dysfunctional self-reactive T cells released from the thymus may persist long-term in both the central and peripheral nervous systems, potentially triggering these autoimmune disorders (19, 20). While thymectomy is a treatment for MG, it may also act as a trigger for PERM by disrupting T-cell regulatory activity. The management of both MG and PERM shares similarities, requiring steroids and immunosuppressants. However, the precise relationship between thymoma, MG, and PERM warrants further investigation.

**Table 2:** Clinical characteristics and outcomes of previously reported cases and the present case.

Ref.	Age/sex	Clinical features	MRI (brain and spinal cord)	EMG	Antibodies	Tumor type	Management	Outcomes
Morise S, et al., 2017 (15)	72/female	Dysarthria, chewing difficulties, myoclonic jerks, stiffness, spasm, dysarthria, myoclonus, hyperreflexia, slow saccade, vertical gaze restriction, horizontal gaze palsy, psychiatric symptoms, myasthenia	Normal	Continuous contraction of both agonist and antagonist muscles in the extremities during myoclonic jerks	Serum GAD, CSF GAD, serum GlyR, CSF GlyR, AChR, TPO, Tg	Thymoma	IVIG, PLEX, corticosteroids, tacrolimus, thymectomy	Good
Ogawa T, et al., 2021 (18)	44/female	Dysphasia, difficulties in swallowing, gait disturbance, tight sensation of legs, ptosis, diplopia, dysarthria, masseter muscle weakness, reduced visual acuity, myoclonus, spasms, bouncy legs, weakness, respiratory failure	Normal	Normal	Serum GlyR, CSF GlyR, AChR, titin	Thymoma	IVIG, tacrolimus, corticosteroids, thymectomy	Pharmacologic remission
Present case	44/female	Numbness, pain, generalized rigidity, painful spasms, myoclonus, jerks, diplopia, painful shoulder joints, horizontal gaze impairment	Normal	Normal	Serum GAD65, CSF GAD65, serum GlyR, CSF GlyR, AChR, titin	Thymoma	IVIG, tacrolimus, corticosteroids, thymectomy	Good

EMG, electromyograph; GAD, glutamic acid decarboxylase; GlyR, glycine receptor; AChR, acetylcholine receptor; CSF, cerebrospinal fluid; Tg, thyroglobulin; TPO, thyroid peroxidase; IVIG, intravenous injection of immunoglobulin; PLEX, plasma exchange.

The coexistence of PERM and MG was observed as a combination of central and peripheral symptoms, including systemic myoclonus and spasms, tachycardia, ptosis, diplopia, slurred speech, dysphasia, and respiratory failure. Given the patient’s history of MG and thymoma, her presentation with myoclonus and spasms during admission prompted further evaluation, as myoclonus is uncommon in MG. Clinicians should be aware that autoimmune diseases can present with overlapping features. If a patient experiences unexpected changes in their condition, exhibits a poor response to initial treatment, or develops new symptoms, the possibility of concurrent autoimmune diseases should be considered. Additionally, the presence of one autoimmune disorder should prompt screening for others to facilitate early diagnosis and intervention.

In conclusion, we report a case of PERM following thymectomy in a middle-aged woman with MG. Autoimmune encephalitis and systemic autoimmune diseases can coexist due to thymoma-induced immune dysregulation, leading to the involvement of multiple antibodies. When unexplained symptoms arise in patients with a history of thymoma, clinicians should consider the possibility of multiple antibody involvement. Further research is needed to clarify the relationship between PERM and MG to develop more comprehensive management strategies.

## Data Availability

The original contributions presented in the study are included in the article/**Supplementary Material**. Further inquiries can be directed to the corresponding author.

## References

[1] Espay AJ Chen R. Rigidity and spasms from autoimmune encephalomyelopathies: stiff-person syndrome. Muscle Nerve. (2006) 34:677–90. doi: 10.1002/mus.20653 16969837

[2] Blum P Jankovic J. Stiff-person syndrome: an autoimmune disease. Mov Disord. (1991) 6:12–20. doi: 10.1002/mds.870060104 2005917

[3] Baizabal-Carvallo JF Jankovic J. Stiff-person syndrome: insights into a complex autoimmune disorder. J Neurol Neurosurg Psychiatry. (2015) 86:840–8. doi: 10.1136/jnnp-2014-309201 25511790

[4] Whiteley AM Swash M Urich H. Progressive encephalomyelitis with rigidity. Brain. (1976) 99:27–42. doi: 10.1093/brain/99.1.27 963529

[5] Carvajal-González A Leite MI Waters P Woodhall M Coutinho E Balint B. Glycine receptor antibodies in perm and related syndromes: characteristics, clinical features and outcomes. Brain. (2014) 137:2178–92. doi: 10.1093/brain/awu142 PMC4107739 24951641

[6] Clardy SL Lennon VA Dalmau J Pittock SJ Jones HR Jr. Renaud DL. Childhood onset of stiff-man syndrome. JAMA Neurol. (2013) 70:1531–6. doi: 10.1001/jamaneurol.2013.4442 PMC4819072 24100349

[7] McKeon A Martinez-Hernandez E Lancaster E Matsumoto JY Harvey RJ McEvoy KM. Glycine receptor autoimmune spectrum with stiff-man syndrome phenotype. JAMA Neurol. (2013) 70:44–50. doi: 10.1001/jamaneurol.2013.574 23090334 PMC3718477

[8] Meinck HM Thompson PD. Stiff man syndrome and related conditions. Mov Disord. (2002) 17:853–66. doi: 10.1002/mds.10279 12360534

[9] Yu H Bai XC Wang W. Characterization of the subunit composition and structure of adult human glycine receptors. Neuron. (2021) 109:2707–16.e6. doi: 10.1016/j.neuron.2021.08.019 34473954

[10] Martinez-Hernandez E Ariño H McKeon A Iizuka T Titulaer MJ Simabukuro MM. Clinical and immunologic investigations in patients with stiff-person spectrum disorder. JAMA Neurol. (2016) 73:714–20. doi: 10.1001/jamaneurol.2016.0133 PMC5020136 27065452

[11] Duarte S Santos E Martins J Martins Silva A Lopes C Gonçalves G. Myasthenia gravis with systemic and neurological polyautoimmunity. J Neurol Sci. (2017) 381:39–40. doi: 10.1016/j.jns.2017.08.010 28991712

[12] Gilhus NE. Myasthenia gravis. N Engl J Med. (2016) 375:2570–81. doi: 10.1056/NEJMra1602678 28029925

[13] Lee HL Min JH Seok JM Cho EB Lee SS Cho HJ. Stiff-person syndrome after thymectomy in myasthenia gravis mimicking a post-thymectomy myasthenic crisis. Neurol India. (2017) 65:1152–3. doi: 10.4103/neuroIndia.NI_493_16 28879920

[14] Nicholas AP Chatterjee A Arnold MM Claussen GC Zorn GL Jr. Oh SJ. Stiff-persons’ Syndrome associated with thymoma and subsequent myasthenia gravis. Muscle Nerve. (1997) 20:493–8. doi: 10.1002/(sici)1097-4598(199704)20:4<493::aid-mus13>3.0.co;2- 9121508

[15] Morise S Nakamura M Morita JI Miyake K Kunieda T Kaneko S. Thymoma-associated progressive encephalomyelitis with rigidity and myoclonus (Perm) with myasthenia gravis. Intern Med. (2017) 56:1733–7. doi: 10.2169/internalmedicine.56.7979 PMC5519481 28674368

[16] Thomas S Critchley P Lawden M Farooq S Thomas A Proudlock FA. Stiff person syndrome with eye movement abnormality, myasthenia gravis, and thymoma. J Neurol Neurosurg Psychiatry. (2005) 76:141–2. doi: 10.1136/jnnp.2004.036558 PMC1739318 15608018

[17] Saravanan PK Paul J Sayeed ZA. Stiff person syndrome and myasthenia gravis. Neurol India. (2002) 50:98–100. 11960163

[18] Ogawa T Ogaki K Daida K Nishimaki T Ando M Kawajiri S. Progressive encephalomyelitis with rigidity and myoclonus and Myasthenia gravis comorbid status with thymoma. Mov Disord Clin Pract. (2021) 8:S11–s3. doi: 10.1002/mdc3.13293 PMC8414492 34514036

[19] Shelly S Agmon-Levin N Altman A Shoenfeld Y. Thymoma and autoimmunity. Cell Mol Immunol. (2011) 8:199–202. doi: 10.1038/cmi.2010.74 21317916 PMC4012878

[20] Ao YQ Jiang JH Gao J Wang HK Ding JY. Recent thymic emigrants as the bridge between thymoma and autoimmune diseases. Biochim Biophys Acta Rev Cancer. (2022) 1877:188730. doi: 10.1016/j.bbcan.2022.188730 35469968

